# Exosomes Derived from Colon Cancer Cells Promote Tumor Progression and Affect the Tumor Microenvironment

**DOI:** 10.3390/jcm12123905

**Published:** 2023-06-07

**Authors:** Minsung Kim, Il Tae Son, Gyoung Tae Noh, So-Youn Woo, Ryung-Ah Lee, Bo Young Oh

**Affiliations:** 1 Department of Surgery, Hallym University College of Medicine, Anyang 14068, Republic of Korea; bongkay4@gmail.com (M.K.); 1tae99@hanmail.net (I.T.S.); 2 Department of Surgery, Ewha Womans University College of Medicine, Seoul 07804, Republic of Korea; nogang@ewha.ac.kr (G.T.N.); ralee@ewha.ac.kr (R.-A.L.); 3 Department of Microbiology, Ewha Womans University College of Medicine, Seoul 03760, Republic of Korea; soyounwoo@ewha.ac.kr

**Keywords:** exosomes, tumor microenvironment, mRNA, microRNA, tumor progression

## Abstract

Cancer-cell-derived exosomes confer oncogenic properties in their tumor microenvironment and to other cells; however, the exact mechanism underlying this process is unclear. Here, we investigated the roles of cancer-cell-derived exosomes in colon cancer. Exosomes were isolated from colon cancer cell lines, HT-29, SW480, and LoVo, using an ExoQuick-TC kit, identified using Western blotting for exosome markers, and characterized using transmission electron microscopy and nanosight tracking analysis. The isolated exosomes were used to treat HT-29 to evaluate their effect on cancer progression, specifically cell viability and migration. Cancer-associated fibroblasts (CAFs) were obtained from patients with colorectal cancer to analyze the effect of the exosomes on the tumor microenvironment. RNA sequencing was performed to evaluate the effect of the exosomes on the mRNA component of CAFs. The results showed that exosome treatment significantly increased cancer cell proliferation, upregulated N-cadherin, and downregulated E-cadherin. Exosome-treated cells exhibited higher motility than control cells. Compared with control CAFs, exosome-treated CAFs showed more downregulated genes. The exosomes also altered the regulation of different genes involved in CAFs. In conclusion, colon cancer-cell-derived exosomes affect cancer cell proliferation and the epithelial–mesenchymal transition. They promote tumor progression and metastasis and affect the tumor microenvironment.

## 1. Introduction

Exosomes are small, membrane-enclosed, extracellular vesicles with a diameter of 30–200 nm secreted by various cell types [1,2]. They are detected in various body fluids, including plasma, ascites, urine, and saliva [3,4]. Exosomes transfer various molecular substances from their originating cells, including lipids, proteins, cytokines, DNA, mRNA, and miRNA [5], to recipient cells and therefore are crucially involved in cell-to-cell interactions [6].

In cancer, exosomes are involved in complex processes during tumor progression. As compared to normal cells, cancer cells secrete higher levels of exosomes [7]. These tumor-derived exosomes deliver oncogenic factors to target cells and the tumor microenvironment. Although the biological characteristics of tumor-derived exosomes remain unclear, they promote cellular proliferation, angiogenesis, and tumor invasion in recipient cells, leading to cancer progression [8,9]. Additionally, tumor-derived exosomes influence the epithelial–mesenchymal transition (EMT) to support metastasis [10]; accordingly, tumor-derived exosomes are termed “oncosomes” [11]. Numerous studies have demonstrated exosomal effects on several cancers, including colon, lung, and breast cancer. However, the molecular and genetic mechanisms underlying the roles of tumor-derived exosomes in cancer remain unclear. Therefore, in this study, we aimed to investigate the roles of cancer-cell-derived exosomes in colon cancer.

## 2. Materials and Methods

### 2.1. Cell Culture

HT-29 (ATCC, HTB-38™) and SW480 (ATCC, CCL-228™) cells were cultured in Roswell Park Memorial Institute medium (WELGENE, Gyeongsan, Republic of Korea; LM001-01). LoVo (ATCC, CCL-229™) cells were cultured in Dulbecco’s Modified Eagle Medium (WELGENE, Republic of Korea) supplemented with 10% fetal bovine serum (WELGNE, S001-011) and 1% penicillin/streptomycin solution 100× (CAPRICORN scientific, Ebsdorfergrund, Germany, PS-B). Cells were grown until they reached 85%–90% confluency. All cell lines were maintained as monolayers at 37 °C in a 5% CO_2_ incubator. All experiments were carried out following relevant guidelines.

### 2.2. Exosome Isolation and Treatment

Exosomes were isolated using the ExoQuick-TC™ exosome precipitation solution (System Biosciences, Palo Alto, CA, USA, EXOTC50A-1) following the manufacturer’s instructions. First, the cell suspension was collected and centrifuged at 300× *g* for 15 min at 4 °C to remove cell debris. The supernatant was transferred to a Macrosep Advance centrifugal device with Omega membrane (Pall, Port Washington, NY, USA) and centrifuged at 5000× *g* for 90 min at 4 °C. Subsequently, a concentrated medium was added to an appropriate volume of ExoQuick-TC precipitation solution. After 12 h, the mixture was centrifuged at 1500× *g* for 30 min at 4 °C. The obtained pellets were resuspended in phosphate-buffered saline (PBS) for the cell treatment and migration assays, as well as in Pro-Prep (iNtRON, 17081) for immunoblotting. Isolated exosomes were immediately used or stored at −80 °C for up to one month.

### 2.3. Immunoblotting

Exosomes were prepared using 1× RIPA buffer (50 mM Tris-HCl, pH 7.6, 150 mM NaCl, 1% Triton-100, 1% sodium deoxycholate, BYLABS, Hanam, Republic of Korea). The exosome concentration was determined using bicinchoninic acid. We added 6X sodium dodecyl sulfate-polyacrylamide gel electrophoresis (SDS-PAGE) loading buffer to prepare the lysate solution. For sample preparation, equal amounts of protein samples were separated using 10% SDS-PAGE and transferred onto 0.45 µm nitrocellulose membranes (GE Healthcare Life Science, Port Washington, NY, USA). The membranes were incubated with 5% skim milk in Tris-buffered saline with 0.1% Tween^®^ 20 Detergent, followed by immunoblotting with specific primary antibodies against CD9 (rabbit monoclonal, Abcam, Cambridge, UK, ab92726), CD81 (mouse monoclonal, sc-166029), glyceraldehyde-3-phosphate dehydrogenase (Santa Cruz, sc-137179, Dallas, TX, USA), E-cadherin (rabbit monoclonal, Abcam, ab92547), β-catenin (rabbit monoclonal, Abcam, ab32576), and N-cadherin (rabbit polyclonal, Abcam, ab18203), followed by appropriate secondary antibodies. Protein bands were visualized using an enhanced chemiluminescence (ECL) substrate (GE Healthcare Life Science) and analyzed using a LAS-3000 imager (GE Healthcare Life Science) and film.

### 2.4. Transmission Electron Microscopy (TEM)

The purified exosomes were diluted to 1:1000 in PBS. We added 5 μL of diluted exosomes into Formvar-carbon-coated TEM grids. The grids were stained using 2% uranyl acetate, which was removed using filter paper. Finally, the grids were viewed using H-7650 TEM (Hitachi, Tokyo, Japan) at an 80 kV voltage. Digital images with a scale bar were captured at 70,000–200,000 magnification.

### 2.5. NanoSight Tracking Analysis (NTA)

NTA was performed using Nanosight NS300 (Malvern Panalytical Ltd., Malvern, UK) to measure the exosome size and concentration in the liquid suspension. Visible EV-containing pellets were resuspended in 100–500 μL of PBS for NTA. For each sample, we recorded five 60 s videos.

### 2.6. MTT Assay

HT-29 cells were plated in a 96-well flat cell culture plate (SPL Life Sciences, Gyeonggi-do, Republic of Korea) at a density of 5 × 10^4^ per well for 24 h at 37 °C in a 5% CO_2_ incubator. Exosomes (100 µg/mL) were isolated from HT-29 and used to treat SW480 and LoVo cells for 24 h. Supernatants from each well were discarded, followed by the addition of 5 mg/mL 3-(4,5-Dimethyl-2-thiazolyl)-2,5-diphenyl-2H-tetrazolium bromide (MTT, methylthiazolyldiphenyl-tetrazolium bromide) solution. After incubation for 4 h, 200 µL of dimethyl sulfoxide (Merck, Darmstadt, Germany) was added. Next, the plates were gently shaken for 15 min at room temperature. The absorbance of each sample was measured at 570 nm using a SpectraMax Versa microplate reader (Molecular Devices, San Jose, CA, USA).

### 2.7. Wound-Healing Assay

We performed a wound-healing assay using SPLScar™Block (SPL Life Sciences, 201935). The block comprised a 500 μm-thick wall to artificially generate a cell-free gap. We treated 100 μg/mL of each cell line with exosomes. The blocks were incubated at 37 °C for 24 h and 48 h.

### 2.8. Isolation and Culture of Cancer-Associated Fibroblasts (CAFs)

Tumor samples were obtained from patients with colorectal cancer who underwent surgery between June 2020 and August 2020 at a university-based hospital. All the patients provided informed consent. The study was approved by the Ewha Medical Center Seoul Hospital Institutional Review Board (IRB No. SEUMC 2019-12-028). CAFs were isolated from the collected tumor samples and cultured as previously described [12].

### 2.9. RNA Sequencing

Exosomes isolated from HT-29, SW480, and LoVo cells were added to CAFs (density 1 × 10^4^) at a concentration of 100 µg/mL for 24 h. Each CAF was collected, followed by the isolation of total RNA using an extraction kit (Qiagen, Hilden, Germany). The RNA sequencing was performed at Macrogen (Seoul, Republic of Korea). The purified RNAs were treated with DNase I to remove genomic DNA contamination. cDNA libraries were prepared using the TruSeq Stranded mRNA LT Sample Prep Kit (Illumina, San Diego, CA, USA) and the amplified libraries were sequenced using the Illumina platform. Trimmed reads were mapped to the reference genome (GRCh38) using HISAT2 v2.1.0 and Bowtie2 v2.3.4.1. After confirming the number of processed and mapped reads, known genes/transcripts were assembled into a reference gene model and normalized to fragments per kilobase of transcript per million mapped reads and transcript per kilobase million using StringTie v2.1.3b. For differentially expressed gene (DEG) analysis, we performed a trimmed mean of M-values (TMM) normalization using the edgeR package in the R library, followed by a comparison of fold changes using the “exactTest” function in edgeR. For data visualization, we used the log_2_ (counts per million reads + 1) and log_2_ (TMM normalized counts + 1) values. Hierarchical clustering analysis was performed using the complete linkage method and the Euclidean distance metric.

### 2.10. Statistical Analysis

Statistical analyses were performed using GraphPad Prism (9.3.1) (GraphPad Software Inc., San Diego, CA, USA). All data are presented as mean ± standard deviation. Comparisons were performed using analysis of variance. Statistical significance was set at *p* < 0.05.

## 3. Results

### 3.1. Isolation of Colon Cancer-Cell-Derived Exosomes

**Figure 1:**
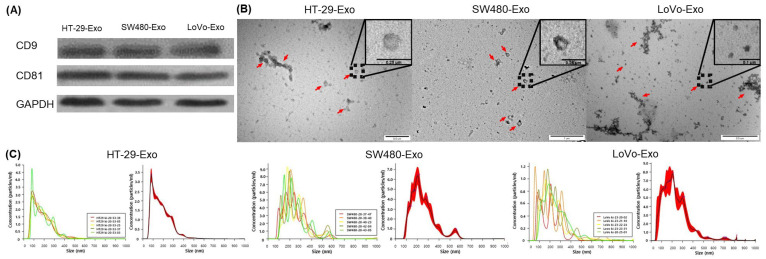
Identification and characterization of colon cancer-cell-derived exosomes. (**A**) Immunoblotting assay for exosome markers detected (CD9 and CD81) in exosomes from HT-29 (HT-29-Exo), SW480 (SW480-Exo), and LoVo cells (LoVo-Exo). (**B**) Exosomes detected using transmission electron microscopy (scale bar, 1 µm). The red arrowhead points toward exosomes. (**C**) HT-29-Exo, SW480-Exo, and LoVo-Exo were detected using Nanosight tracking analysis. The left column represents batch-to-batch variations, while the right column shows the overall size of HT-29-Exo, SW480-Exo, and LoVo-Exo. The mean sizes of HT-29-Exo, SW480-Exo, and LoVo-Exo were 178.3 ± 1.3 nm, 250.9 ± 3.4 nm, and 229.7 ± 8.7 nm, respectively, while their mean concentrations were 4.95 × 10^9^ ± 4.41 × 10^7^, 1.20 × 10^9^ ± 1.82 × 10^7^, and 1.86 × 10^9^ ± 1.22 × 10^7^ particles/mL, respectively.

Exosomes were isolated from cultured colon cancer cell lines (HT-29 [HT-29-Exo], SW480 [SW480-Exo], and LoVo [LoVo-Exos]) using ExoQuick-TC™. The extracted exosomes were confirmed using Western blotting for exosome markers (CD9 and CD81; Figure 1A). The exosomal structures were determined using TEM (Figure 1B). NTA was performed to characterize the size and number of nanoparticles isolated from each cell line. The curves show that most isolated vesicles were exosomes (Figure 1C).

### 3.2. Cellular Proliferation Induced by Colon Cancer-Cell-Derived Exosomes

**Figure 2:**
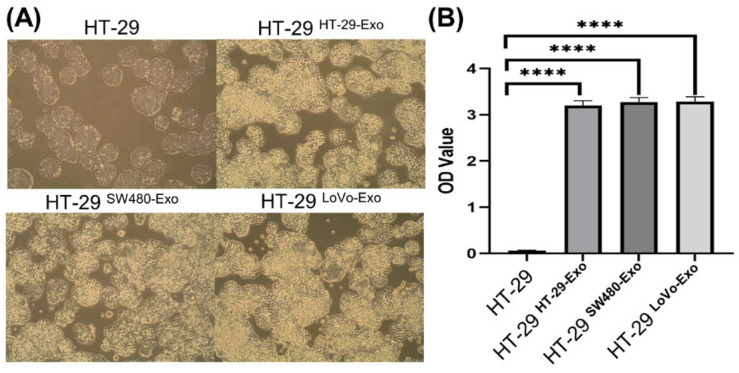
Proliferative effect of exosomes derived from colon cancer cells. HT-29 cells were treated with HT-29-Exo, SW480-Exo, and LoVo-Exo at the same concentration of 100 µg/mL for 24 h, and the proliferation was assessed. (**A**) All three exosomes increased the cellular proliferation of the HT-29 cells. (**B**) In the MTT assay, the exosome-treated HT-29 cells showed higher viability than the untreated control cells. Bars represent mean ± standard deviation (SD); *n* = 3; **** *p* < 0.0001.

We treated HT-29 cells with HT-29-Exo, SW480-Exo, and LoVo-Exo to investigate their effect on tumor growth. Compared to the untreated control cells, the exosome-treated cells showed significantly increased growth (Figure 2A). Moreover, the MTT assay revealed the significantly increased proliferation of the exosome-treated cells compared to that of the untreated control cells (Figure 2B).

### 3.3. EMT Induction by Colon Cancer-Cell-Derived Exosomes

**Figure 3:**
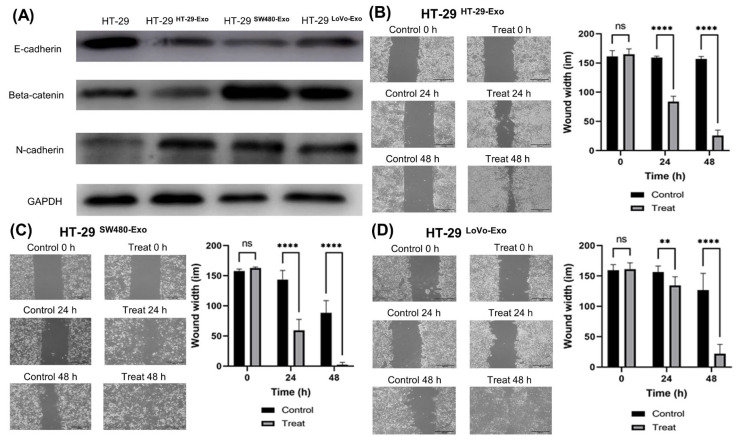
EMT induction by exosomes derived from colon cancer cells. (**A**) E-cadherin, beta-catenin, and N-cadherin expression analyzed in exosome-treated HT-29 cells. Glyceraldehyde-3-phosphate dehydrogenase was used as the loading control. (**B**–**D**) Representative images of wound-healing assay performed after treatment with cancer-cell-derived exosomes. The bar graphs on the right show wound width. Data show mean ± SD (*n* = 3). ** *p* < 0.01, **** *p* < 0.0001.

Western blotting for EMT markers revealed E-cadherin downregulation and β-catenin and N-cadherin upregulation in the exosome-treated cells (Figure 3A). Considering that the migration of cancer cells is essential for EMT during metastasis, we further investigated the mobility properties and malignancy potential of these cells. The wound-healing assay showed that the exosome-treated cells had greater mobility properties and malignancy potential than the control cells (Figure 3B–D).

### 3.4. RNA Sequencing Analysis of CAFs Treated with Colon Cancer Cell-Derived Exosomes

**Figure 4:**
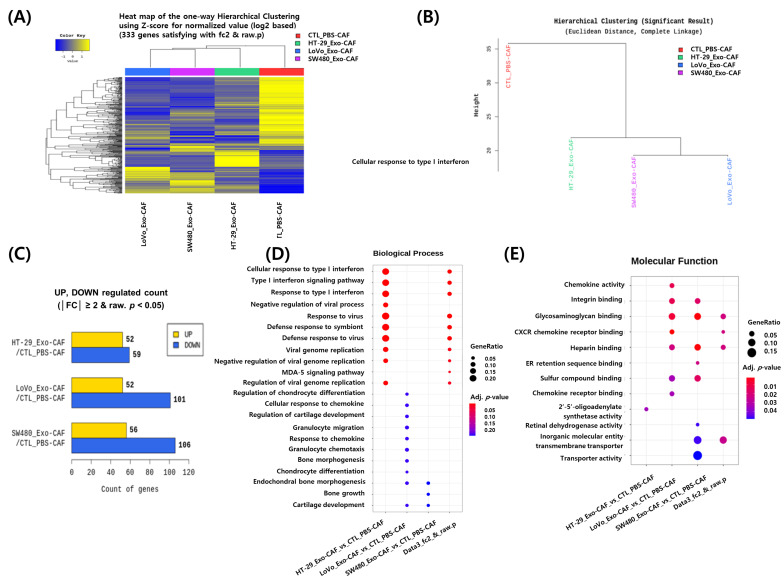
RNA sequencing data analysis of PBS-treated Cancer-associated fibroblasts (CAFs) (PBS-CAF), HT-29-Exo-treated CAFs (HT-29_Exo-CAF), SW480-Exo-treated CAFs (SW480_Exo-CAF), and LoVo-Exo-treated CAFs (LoVo_Exo-CAF). (**A**) Heatmap of hierarchical clustering showing differentially expressed genes (rows) among CAFs treated with exosomes from different sources. Yellow and blue indicate upregulated and downregulated genes, respectively. (**B**) Dendrogram of hierarchical clustering indicates the similarities in gene expression among the exosome-treated CAFs. (**C**) Differentially expressed genes between control and exosome-treated CAFs. The yellow and blue bars indicate the number of upregulated and downregulated genes in exosome-treated CAFs compared with control CAFs, respectively. (**D**,**E**) Functional enrichment analysis of highly regulated genes among CAFs in the five experimental groups. Distribution of gene ontology (GO) terms of DEG was annotated in terms of biological processes and molecular functions. All colored circles indicate statistical significance (*p* < 0.05). The size of the circles indicates the between-group gene ratio.

The tumor microenvironment is crucially involved in tumor progression by interacting with cancer cells. We investigated the involvement of exosomes in these interactions and their influence on the tumor microenvironment. Therefore, we isolated CAFs, which are major stromal cells, from tumor samples and treated them with cancer-cell-derived exosomes. Subsequently, we analyzed changes in the mRNA components of CAFs after treatment with exosomes. After cDNA mapping to the reference genome, an average of 76.2 million 100-base long reads were obtained from the CAFs. To examine transcriptome differences between exosome-treated and untreated control CAFs, a heatmap was generated using the Z-score for normalized log_2_ values for 333 genes (Figure 4A). A dendrogram was generated to determine differences among the different exosome sources (Figure 4B). The RNA expression profiles of the four exosome-treated CAFs significantly differed from that of the wild CAF, with LoVo-Exo-treated CAFs showing the largest difference. As shown in Figure 4C, the number of downregulated genes was higher than that of upregulated ones in exosome-treated CAFs. The functional enrichment analysis of highly regulated genes according to gene ontology (GO) terms of DEGs revealed that HT-29-Exo altered the expression of genes associated with viral infection, including the type I interferon pathway. The treatment with LoVo-Exo altered the expression of those involved in cell migration, including chemokine and granulocyte migration, wherein SW480-Exo altered the expression of genes related to bone and cartilage development (Figure 4D). The molecular function annotation analysis revealed the association of SW480-Exo with transporter activity (Figure 4E).

## 4. Discussion

Cancer-cell-derived exosomes play crucial roles in cancer progression and metastasis [8,10]. In this study, we demonstrated that exosomes derived from colon cancer cells enhanced tumor cell proliferation and EMT induction. Furthermore, these exosomes also affected the tumor microenvironment. Taken together, our results suggest that cancer-cell-derived exosomes alter RNA expression, gene regulation, and gene expression in tumor cells.

The methodology employed for exosome isolation generally involves a series of differential ultracentrifugation procedures, typically 100,000× *g* for 2–3 h. Further purification of exosomes can be achieved by floating them on a continuous sucrose density gradient [6,13]. These isolation techniques enable the enrichment of exosomes from the cell culture medium. In this study, we used a polymer precipitation-based method that is simple, rapid, and scalable for large sample sizes [6,12,13]. Exosomes derived from various cell types share a common set of proteins, indicating their conserved composition. These proteins include members of the tetraspanin family (CD9, CD63, CD81, and CD82), components of the endosomal sorting complexes required for the transport machinery complex (TSG101, Alix), heat-shock proteins (Hsp60, Hsp70, and Hsp90), cytoskeletal proteins (myosin, β-actin, and tubulin), integrins, fusion proteins (annexins, Rab GTPases, flotillins), and glycolytic proteins (enolase 1 and glyceraldehyde 3-phosphate dehydrogenase) [10,12]. In this study, exosomes were isolated from the cultured colon cancer cell lines HT-29, SW480, and LoVo. The confirmation of their identity as exosomes was based on the presence of the surface markers CD9 and CD81, as well as size measurements obtained using TEM and NTA. TEM is widely used to visualize extracellular vesicles and delineate their structure, morphology, and size [6,12]. Like other extracellular vesicles, the morphology of exosomes derived from cancer cells can be determined using electron microscopy. These exosomes are characterized as spherical membrane-bound vesicles, typically measuring less than 50 nm in diameter [6].

Exosomes released from cancer cells contain various proteins and nucleic acids, which promote tumor progression through interactions with other tumor cells and the tumor microenvironment [1,4,8]. The transfer of oncogenes and oncogenic signals were transferred through exosomes in paracrine communication, either from one tumor cell to another or from one tumor cell to normal cells [6,11,14]. Some studies have demonstrated that the K-ras mutant type of colon cancer cell releases exosomes that carry mutant K-ras and various growth-promoting proteins such as EGFR, Src family kinases, and integrins [15]. Moreover, exosomes derived from cancer cells include activated oncoprotein, mRNA, oncogenic DNA sequences, and oncogenic microRNAs, which can modify the expression of genes involved in critical cellular processes, including the production of growth factors [6,11,16]. For instance, activated oncoproteins, which are proteins with the potential to promote cancer progression, can influence cellular signaling pathways [6]. mRNA can be transferred and translated in recipient cells, leading to the production of these oncogenic proteins. Oncogenic DNA sequences can be transmitted to recipient cells, integrating into their genome and altering the genetic information, leading to the dysregulation of genes involved in cellular growth, and thus contributing to the progression of tumor cells. Moreover, oncogenic microRNAs can be transferred between cells. In our study, exosome treatment significantly increased cellular proliferation. Concordant with our findings, those of a previous study have shown that the Wnt5b protein carried by exosomes promotes cancer cell proliferation in a paracrine manner [17]. Furthermore, mRNAs carried by exosomes are crucially involved in cellular activities, including cellular proliferation and migration. Over 11,000 distinct mRNAs in exosomes derived from colon cancer cells affect cancer cell proliferation [18]. In addition, exosomal miRNAs are associated with tumor progression. miR-21 enriched with exosomes derived from colon cancer cells could inhibit PDCD4 protein translation involved in cell apoptosis [19]. Heat-shock proteins, which are increased in exosomes due to cellular stresses, can inhibit cell apoptosis and promote cell proliferation [20]. Additionally, a hypoxic environment can promote exosome secretion by cancer cells as well as cellular proliferation through STAT3 activation and shortening the mitosis duration [4].

EMT is a hallmark of aggressive tumors and is the primary mechanism underlying tumor metastasis [1,8,21]. Cancer-cell-derived exosomes can induce EMT [22]. Exosomes derived from colon cancer cells containing high miR-210 levels are involved in inducing EMT, which results in a metastatic phenotype [1,23]. The hallmark of EMT is N-cadherin upregulation followed by E-cadherin downregulation [24,25]. β-catenin activation induces EMT progression by modifying cell–cell junctions [26]. In our study, Western blotting revealed E-cadherin downregulation and β-catenin and N-cadherin upregulation in the exosome-treated cells, suggesting that cancer-cell-derived exosomes promote EMT. In the wound-healing assay, the exosome-treated cells showed greater malignancy potential than the control cells. Cell-to-cell communication via exosomes between primary cancer cells and the microenvironment of distant organs is crucial for premetastatic niche formation [27,28]. An in vivo study reported that mRNA carried by cancer-cell-derived exosomes affects the migration and metastasis of primary and distant tumor cells [29]. Cancer-cell-derived exosomes are significantly involved in manipulating the tumor microenvironment to form a pro-tumorigenic soil [10]. In addition, cancer-cell-derived exosomes target non-transformed cells in premetastatic organs and modulate premetastatic organ cells predominantly through transferred miRNA; specifically, miRNA from a metastasizing tumor prepares the stromal cells of the premetastatic organ for tumor cell hosting [30].

CAFs are crucial components of the tumor microenvironment that can interact with cancer cells to promote tumor metastasis and progression [31,32]. Cancer-cell-derived exosomes can induce changes from normal fibroblasts to CAFs [33]. In our study, cancer-cell-derived exosomes altered RNA expression, gene regulation, and gene expression in CAFs. CAFs are characterized by their spindle-shaped morphology and express specific markers such as α-smooth muscle actin, fibroblast activation protein-α (FAP-α), fibroblast-specific protein-1, and platelet-derived growth factor receptor -α, and -β. In this study, CAFs were obtained through enzymatic digestion followed by the removal of floating cells after 48 h while preserving the adherent cells expressing FAP-α [34]. It is important to note that when isolating CAFs from colon cancer cells, contamination is possible at the source or during the cell preparation process [12]. In this study, these cell lines were handled separately, preferably in a quarantine setting, to minimize any potential contamination and ensure the integrity of the experimental results. RNA in cancer-cell-derived exosomes can facilitate cancer cell proliferation and migration by reprogramming normal fibroblasts into tumor-promoting CAFs [31,33]. RNA expression, gene regulation, and gene expression differed according to the exosomes used for treatment, which suggests that the RNAs might differ across the types of cancer cells.

This study has several limitations. First, we did not elucidate the specific pathways underlying the effect of cancer-cell-derived exosomes on cancer progression. Second, this study consisted of only in vitro experiments. Third, this study utilized only three different colon cancer cell lines, HT-29, SW480, and LoVo. Initially, in the experimental planning, we selected the cell lines HT-29, a K-ras wild type of primary colon cancer cell; SW480, a K-ras mutant type of primary colon cancer cell; Colo205, a K-ras wild type of metastatic colon cancer cell; and LoVo, K-ras mutant type of metastatic colon cancer cell. However, we failed to culture Colo205 several times, so we experimented with the remaining three cell lines. The K-ras wild type of other metastatic colon cancer cells was not available. Despite these limitations, our findings demonstrate that exosomes derived from various colon cancer cells have common effects on cancer progression, with differences in the underlying biological processes or molecular functions. These findings may assist in easy and early diagnosis and targeted therapies using exosomes in the future.

In conclusion, cancer-cell-derived exosomes affected cancer cell proliferation and EMT. Our findings suggest that cancer-cell-derived exosomes are crucially involved in tumor progression and metastasis and affect the tumor microenvironment. Future studies are warranted to validate the differences in the RNA sequencing values and to determine the in vivo effects of exosomes on cancer progression.

## Data Availability

The datasets generated and analyzed during the current study are available in the Zenodo repository, https://doi.org/10.5281/zenodo.6839155, accessed on 15 July 2022.
